# Expression and clinical value of programmed cell death-ligand 1 (PD-L1) in diffuse large B cell lymphoma: a retrospective study

**DOI:** 10.1186/s40880-017-0262-z

**Published:** 2017-12-16

**Authors:** Li-Yang Hu, Xiao-Lu Xu, Hui-Lan Rao, Jie Chen, Ren-Chun Lai, Hui-Qiang Huang, Wen-Qi Jiang, Tong-Yu Lin, Zhong-Jun Xia, Qing-Qing Cai

**Affiliations:** 1 State Key Laboratory of Oncology in South China, Collaborative Innovation Center of Cancer Medicine, Sun Yat-sen University Cancer Center, Guangzhou, 510060 Guangdong P. R. China; 2 Department of Medical Oncology, Sun Yat-sen University Cancer Center, Guangzhou, 510060 Guangdong P. R. China; 3 Department of Medical Oncology, The Fifth Affiliated Hospital of Sun Yat-sen University, Guangzhou, 519000 Guangdong P. R. China; 4 Department of Pathology, Sun Yat-sen University Cancer Center, Guangzhou, 510060 Guangdong P. R. China; 5 Guangdong Province Key Laboratory of Arrhythmia and Electrophysiology, Guangzhou, 510120 Guangdong P. R. China; 6 Department of Radiotherapy, Sun Yat-sen Memorial Hospital of Sun Yat-sen University, Guangzhou, 510120 Guangdong P. R. China; 7 Department of Anesthesiology, Sun Yat-sen University Cancer Center, Guangzhou, 510060 Guangdong P. R. China; 8 Department of Hematology Oncology, Sun Yat-sen University Cancer Center, Guangzhou, 510060 Guangdong P. R. China

**Keywords:** Programmed cell death-ligand 1 (PD-L1), Diffuse large B-cell lymphoma, C-Myc, Prognosis

## Abstract

**Background:**

The programmed cell death-1 (PD-1)/programmed cell death-ligand 1 (PD-L1) pathway inhibits the activation of T cells and plays a crucial role in the negative regulation of cellular and humoral immune responses. Diffuse large B-cell lymphoma (DLBCL) is the most common lymphoid malignancy in adults. In the present study, we aimed to detect the expression of PD-L1 in DLBCL and to analyze its relationship with prognosis.

**Methods:**

We reviewed medical records of 204 newly diagnosed DLBCL patients in Sun Yat-sen University Cancer Center between October 2005 and August 2012. The expression of PD-L1 in tumor tissues from these 204 patients was detected using immunohistochemical (IHC) assay. The expression of anaplastic lymphoma kinase (ALK), CD5, CD30, and C-Myc in tumor specimens from 109 patients was detected using IHC, and Epstein–Barr virus (EBV)-encoded RNAs (EBERs) were detected using fluorescence in situ hybridization. The Spearman method was used for correlation analysis. The Kaplan–Meier method with log-rank test was used for univariate analysis. Cox proportional hazards model was used for multivariate analysis.

**Results:**

Of the 204 patients, 100 (49.0%) were PD-L1-positive in tumor cells and 44 (21.6%) were PD-L1-positive in tumor microenvironment. PD-L1 expression in tumor cells and tumor microenvironment were more common in the non-germinal center B-cell-like (GCB) subtype than in the GCB subtype (*P* = 0.02 and *P* = 0.04). Patients with PD-L1 expression in tumor microenvironment were more likely to be resistant to first-line chemotherapy when compared with the patients without PD-L1 expression in tumor microenvironment (*P* = 0.03). PD-L1 expression in tumor microenvironment was negatively correlated with C-Myc expression (*r* = − 0.20, *P* = 0.04). No correlations were detected between PD-L1 expression and the expression of ALK, CD5, and CD30 as well as EBERs. The 5-year overall survival (OS) rates were 50.0% and 67.3% in patients with and without PD-L1 expression in tumor cells (*P* = 0.02). PD-L1 expression in tumor cells was an independent risk predictor for OS (*P* < 0.01).

**Conclusions:**

PD-L1 expression is more common in the non-GCB subtype than in the GCB subtype. PD-L1 expression in tumor microenvironment has a negative correlation with C-Myc. PD-L1 positivity predicts short survival in DLBCL patients. For patients with PD-L1 expression, more strategy such as anti-PD-L1 antibody treatment should be recommended.

## Background

Programmed cell death-ligand 1 (PD-L1), a member of the B7 family (also known has B7-H1), is an inhibitory ligand expressed on the surface of macrophages, dendritic cells, fibroblasts, and T cells [1, 2]. Binding of PD-L1 to its receptor, programmed cell death 1 (PD-1), inhibits cytokine production and cell cycle progression of T cells [3–5]. It functions as an important checkpoint in the regulation of cellular and humoral immune responses [6]. Adaptive immune responses that include PD-1/PD-L1 expression are associated with cancer relapse [7]. PD-1/PD-L1 is an important axis that has important roles in the infiltration of various immune effectors and in the propensity to develop metastatic disease. Recent evidence suggests that activation of the PD-1/PD-L1 pathway represents one mechanism that allows tumors to elude the host immune system [8]. Previous studies have reported that PD-L1 is involved in the negative regulation of immune responses by binding to the PD-1 receptor and results in cancer cells evading the host immune surveillance and the promotion of metastasis [9].

Aberrant PD-L1 expression was reported in a number of human malignancies [1, 7, 10]. Evidence suggests that PD-L1 expression is associated with prognosis of certain types of cancers [11–16]. The PD-1/PD-L1 axis has attracted wide attention in the treatment of cancers such as lymphoma [17–19]. It has been proposed that immunotherapy can be combined with targeted therapy [4, 6]. Therapeutic PD-1/PD-L1 checkpoint inhibitors target the PD-1/PD-L1 immune checkpoint to restore the cancer cell-directed immune response [2, 20, 21]. Monoclonal antibodies directed against the PD-1/PD-L1 axis have shown clinical activity in several solid tumors including ovarian cancer [21], melanoma [22], renal cell carcinoma [23, 24], lung cancer [25], and colorectal cancer [26]. The use of a signaling inhibitor to reduce PD-L1 expression together with anti-PD-1 antibodies showed a promising durable effect against malignant diseases [27, 28].

As a group of heterogeneous diseases, diffuse large B-cell lymphoma (DLBCL) can be divided into different clinical and pathological subtypes as well as molecular and immunophenotypic subgroups [29, 30]. Anaplastic lymphoma kinase (ALK) [31], CD5 [32], CD30 [33], C-Myc [34], and Epstein–Barr virus (EBV)-encoded RNAs (EBERs) [35] are all important markers expressed in DLBCL that may predict prognosis. PD-L1 is also expressed on DLBCL tumor cells and tumor-infiltrating non-malignant cells, primarily macrophages [36]. Macrophages constitute a major source of PD-L1 expression in the tumor microenvironment of lymphomas. Interactions between tumor cells and the immune system are critical in defining disease biology in B-cell lymphomas [37, 38].

It is suggested that coordinate regulation of PD-L1 among tumor cells and tumor-infiltrating macrophages may exist [36]. Research revealed that in B-cell malignancies, the tumor microenvironment releases survival and proliferation signals and contributes to disease progression, drug resistance, and disease relapse [39]. The PD-1/PD-L1 signaling axis plays a critical role in patients with a large amount of tumor-infiltrating macrophages and is related with an inferior clinical outcome through the suppression of anti-tumor immunity [36].

Several studies have reported the expression of PD-L1 in lymphoma and described its relationship with prognosis [5, 40, 41]. However, similar studies on PD-L1 expression in DLBCL in China are rare. The clinicopathological characteristics of PD-L1-positive DLBCL patients are still controversial. In the present study, we retrospectively assessed the expression of PD-L1 in DLBCL tissues and analyzed its association with clinicopathological features and prognosis of patients with DLBCL.

## Patients and methods

### Patient selection

Clinical data were collected from the database of the Department of Medical Oncology, Sun Yat-sen University Cancer Center (Guangzhou, China).

The inclusion criteria were as follows: (1) patients with newly diagnosed DLBCL treated at the Sun Yat-sen University Cancer Center between October 2005 and August 2012; (2) patients were diagnosed using biopsy according to the 2001 or 2008 World Health Organization classification; (3) patients were 18 years of age or older; (4) patients had received first-line chemotherapy regimens, such as R-CHOP (rituximab, cyclophosphamide, doxorubicin, vincristine, and prednisone) or R-CHOP-like regimen, CHOP (cyclophosphamide, doxorubicin, vincristine, and prednisone) or CHOP-like regimen, and MA regimen (high-dose methotrexate and cytarabine) with curative intent; (5) patients were not infected with human immunodeficiency virus and were not with immunodeficiency disease or second tumor; and (6) complete clinical information was available, including follow-up data. The Ann-Arbor staging system and the international prognostic index (IPI) were used for staging evaluation and risk stratification respectively.

### Immunohistochemical assay

All tumor specimens were obtained by biopsy or primary tumor resection before chemotherapy, fixed in 10% buffered formaldehyde, and embedded in paraffin. The 5-μm thick, formalin-fixed, and paraffin-embedded sections were mounted on slides. Slides were deparaffinized in a microwave oven with xylene, then washed using alcohol washes of decreasing concentrations (e.g., 100%, 95%, 80%, 50%) and distilled water. A heat-induced antigen retrieval method was used with sodium citrate buffer solution (pH 8.0). Rabbit monoclonal antibody for PD-L1 (1:50 dilution) was from Cell Signaling Technology (Boston, MA, USA). Mouse monoclonal antibody for other markers assessed in the present study, including ALK (1:50 dilution), CD5 (1:100 dilution), CD30 (1:50 dilution), C-Myc (1:50 dilution), were from Zhongshan Jinqiao Biotechnology (Beijing, China).

Slides were incubated with primary antibodies at 37 °C for 50 min, incubated with secondary antibody at 37 °C for 30 min, and counterstained with hematoxylin. Next, the slides were dehydrated and covered as per routine laboratory protocols. Two independent pathologists reviewed all specimens separately, and a common consensus was reached in all cases. Cases with any tumor cells expressing CD5 or ALK were considered CD5- or ALK-positive; cases with at least 30% of tumor cells expressing C-Myc were considered C-Myc-positive; cases with at least 20% of tumor cells expressing CD30 were considered CD30-positive. Cases with at least 5% of lymphoma cells showing distinct membranous and/or cytoplasmic staining of PD-L1 were considered PD-L1-positive in tumor cells. Cases with at least 20% of all cells (malignant and non-malignant cells) expressing PD-L1 were considered PD-L1-positive in tumor microenvironment. The results of BCL2 and BCL6 expression were collected from clinical records of the patients.

### Fluorescence in situ hybridization

EBERs were detected using the peptide nucleic acid fluorescence in situ hybridization detection kit (Zhongshan Jinqiao) following the manufacturer’s protocol. When at least 10% of cells were stained in the nuclei, the case was defined as EBV-positive.

### Follow-up

After treatment, patients were followed up every 3 months in the first 3 years and every 6 months thereafter. The final date of follow-up was March 31st, 2016. Routine examinations including physical examination, standard laboratory tests, echocardiography, and a whole-body computed tomography (CT) scan or fluorodeoxyglucose positron emission tomography (FDG-PET) were performed during follow-up. Overall survival (OS) was defined as the duration from pathological diagnosis to death or the last follow-up. Progression-free survival (PFS) was defined as the duration from pathological diagnosis to death, disease progression, or the last follow-up. Patients without any event at the last visit were censored.

### Statistical analysis

Chi square test or Fisher’s exact test was used to analyze the relationship between PD-L1 expression in tumor cells/tumor microenvironment and clinicopathological characteristics of DLBCL patients. Pearson’s test was used to analyze the correlation between indexes. The Kaplan–Meier method was used to establish survival curves, and log-rank test was used for univariate analyses. The Cox proportional hazards model was used for multivariate analysis. All statistical analyses used 0.05 as the significance level (two-sided test) and were performed by using SPSS version 19.0 (IBM, Armonk, NY, USA).

## Results

### Patient characteristics

Clinical data of 204 patients with DLBCL were included in our analysis. The detailed baseline characteristics according to PD-L1 expression in tumor cells and tumor microenvironment are shown in Table 1. Among these patients, 115 (56.4%) were male, and 89 (43.6%) were female. The median age was 52 years (range 18–86 years). One hundred and one (49.5%) patients received R-CHOP/R-CHOP-like regimen as first-line treatment, 97 (47.5%) patients received CHOP/CHOP-like regimen, and 6 (2.9%) patients with central nervous system involvement received high-dose methotrexate and cytarabine. The median number of chemotherapy cycles was 6 (range 1–9). Overall, 104 (51.0%) patients had advanced stage (stages III and IV) disease; 61 (29.9%) had GCB subtype, and 138 (67.6%) had non-GCB-subtype disease. There were 100 (49.0%) patients with PD-L1 expression in tumor cells and 44 (21.6%) with PD-L1 expression in tumor microenvironment. With a median follow-up of 52 months (range 1–114 months), 98 (48.0%) patients experienced disease progression or died.

**Table 1:** Clinicopathological characteristics of 204 patients with diffuse large B-cell lymphoma (DLBCL) according to the expression of programmed cell death-ligand 1 **(**PD-L1) in tumor cells and tumor microenvironment

Characteristic	PD-L1 expression in tumor cells [cases (%)]		*P* value	PD-L1 expression in tumor microenvironment [cases (%)]		*P* value
	Negative	Positive		Negative	Positive	
Total	104	100		160	44	
Gender			0.22			0.34
Male	63 (60.6)	52 (52.0)		93 (58.1)	22 (50.0)	
Female	41 (39.4)	48 (48.0)		67 (41.9)	22 (50.0)	
Age (years)			0.78			0.92
≤ 60	73 (70.2)	72 (72.0)		114 (71.2)	31 (70.5)	
> 60	31 (29.8)	28 (28.0)		46 (28.8)	13 (29.5)	
Clinical stage			0.04			0.12
I–II	58 (55.8)	42 (42.0)		83 (51.9)	17 (38.6)	
III–IV	46 (44.2)	58 (58.0)		77 (48.1)	27 (61.4)	
B symptoms			0.74			0.22
No	75 (72.1)	70 (70.0)		117 (73.1)	28 (63.6)	
Yes	29 (27.9)	30 (30.0)		43 (26.9)	16 (36.4)	
Spleen involvement			0.19			0.22
No	92 (88.5)	82 (82.0)		139 (86.9)	35 (79.5)	
Yes	12 (11.5)	18 (18.0)		21 (13.1)	9 (20.5)	
IPI			0.09			0.18
< 3	86 (82.7)	73 (73.0)		128 (80.0)	31 (70.5)	
≥ 3	18 (17.3)	27 (27.0)		32 (20.0)	13 (29.5)	
Extranodal involvement			0.67			0.88
No	42 (40.4)	44 (44.0)		67 (41.9)	19 (43.2)	
Yes	62 (59.6)	56 (56.0)		93 (58.1)	25 (56.8)	
Bulky disease^a^			0.91			0.82
No	56 (70.0)	63 (69.2)		92 (69.2)	27 (71.1)	
Yes	24 (30.0)	28 (30.8)		41 (30.8)	11 (28.9)	
LDH^b^ (U/L)			0.25			0.27
≤ 245	55 (55.6)	44 (47.3)		81 (53.6)	18 (43.9)	
> 245	44 (44.4)	49 (52.7)		70 (46.4)	23 (56.1)	
ALP^c^ (U/L)			0.17			0.42
≤ 110	93 (92.1)	86 (86.0)		138 (87.9)	41 (93.2)	
> 110	8 (7.9)	14 (14.0)		19 (12.1)	3 (6.8)	
β2-MG^d^ (mg/L)			0.06			0.01
≤ 2.52	25 (69.4)	21 (48.8)		41 (66.1)	5 (29.4)	
> 2.52	11 (30.6)	22 (51.2)		21 (33.9)	12 (70.6)	
Ki-67^e^ (%)			0.74			0.75
< 90	45 (61.6)	45 (64.3)		70 (63.6)	20 (60.6)	
≥ 90	28 (38.4)	25 (35.7)		40 (36.4)	13 (39.4)	
GCB subtype^f^			0.02			0.04
Non-GCB	61 (61.6)	77 (77.0)		102 (65.8)	36 (81.8)	
GCB	38 (38.4)	23 (23.0)		53 (34.2)	8 (18.2)	
Rituximab^g^			0.62			0.10
No	40 (43.0)	42 (46.7)		59 (41.5)	23 (56.1)	
Yes	53 (57.0)	48 (53.3)		83 (58.5)	18 (43.9)	
First-line chemotherapy CR^h^			0.61			0.15
No	31 (33.7)	31 (36.9)		46 (32.6)	16 (45.7)	
Yes	61 (66.3)	53 (63.1)		95 (67.4)	19 (54.3)	
First-line chemotherapy resistance^i^			0.36			0.03
No	83 (90.2)	72 (85.7)		128 (90.8)	27 (77.1)	
Yes	9 (9.8)	12 (14.3)		13 (9.2)	8 (22.9)	
Relapse			0.12			0.93
No	89 (85.6)	77 (77.0)		130 (81.2)	36 (81.8)	
Yes	15 (14.4)	23 (23.0)		30 (18.8)	8 (18.2)	
Death			0.09			0.34
No	69 (66.3)	55 (55.0)		100 (62.5)	24 (54.5)	
Yes	35 (33.7)	45 (45.0)		60 (37.5)	20 (45.5)	

*IPI* international prognostic index, *LDH* lactate dehydrogenase, *ALP* anaplastic lymphoma kinase, *β2-MG* β2-microglobulin, *GCB* germinal center B-cell-like, *CR* complete remission

^a^The data of 33 patients were missing

^b^The data of 12 patients were missing

^c^The data of 3 patients were missing

^d^The data of 125 patients were missing

^e^The data of 61 patients were missing

^f^The data of 5 patients were missing

^g^The data of 21 patients were missing

^h^The data of 28 patients were missing

^i^The data of 28 patients were missing

The expression statuses of ALK, CD5, CD30, C-Myc, and EBERs were only detected in samples from 109 patients because of the insufficient amount of pathological specimens. ALK was only detected in 1 (0.9%) patient; CD5 was positive in 9 (8.3%) patients; CD30 was positive in 16 (14.7%) patients; C-Myc was positive in 18 (16.5%) patients; C-Myc and BCL2 co-expression was observed in 17 (15.6%) patients; and C-Myc and BCL6 co-expression was observed in 9 (8.3%) patients; 9 (8.3%) patients were EBER-negative.

### Relationship of PD-L1 with clinicopathologic characteristics

PD-L1 expression in tumor cells was associated with the non-GCB subtype (*P* = 0.02). PD-L1 expression in tumor microenvironment was associated with the non-GCB subtype (*P* = 0.04), elevated β2-MG level (*P* = 0.01), and resistance to first-line chemotherapy (*P* = 0.03). The expression level of PD-L1 in tumor microenvironment had a negative correlation with that of C-Myc (*r* = − 0.20, *P* = 0.04). No correlation of the expression level of PD-L1 in tumor cells with that of C-Myc was observed. There was no significant association of PD-L1 expression in either tumor cells or microenvironment with CD5, CD30, C-Myc, and EBERs.

### Survival analysis

**Fig. 1:**
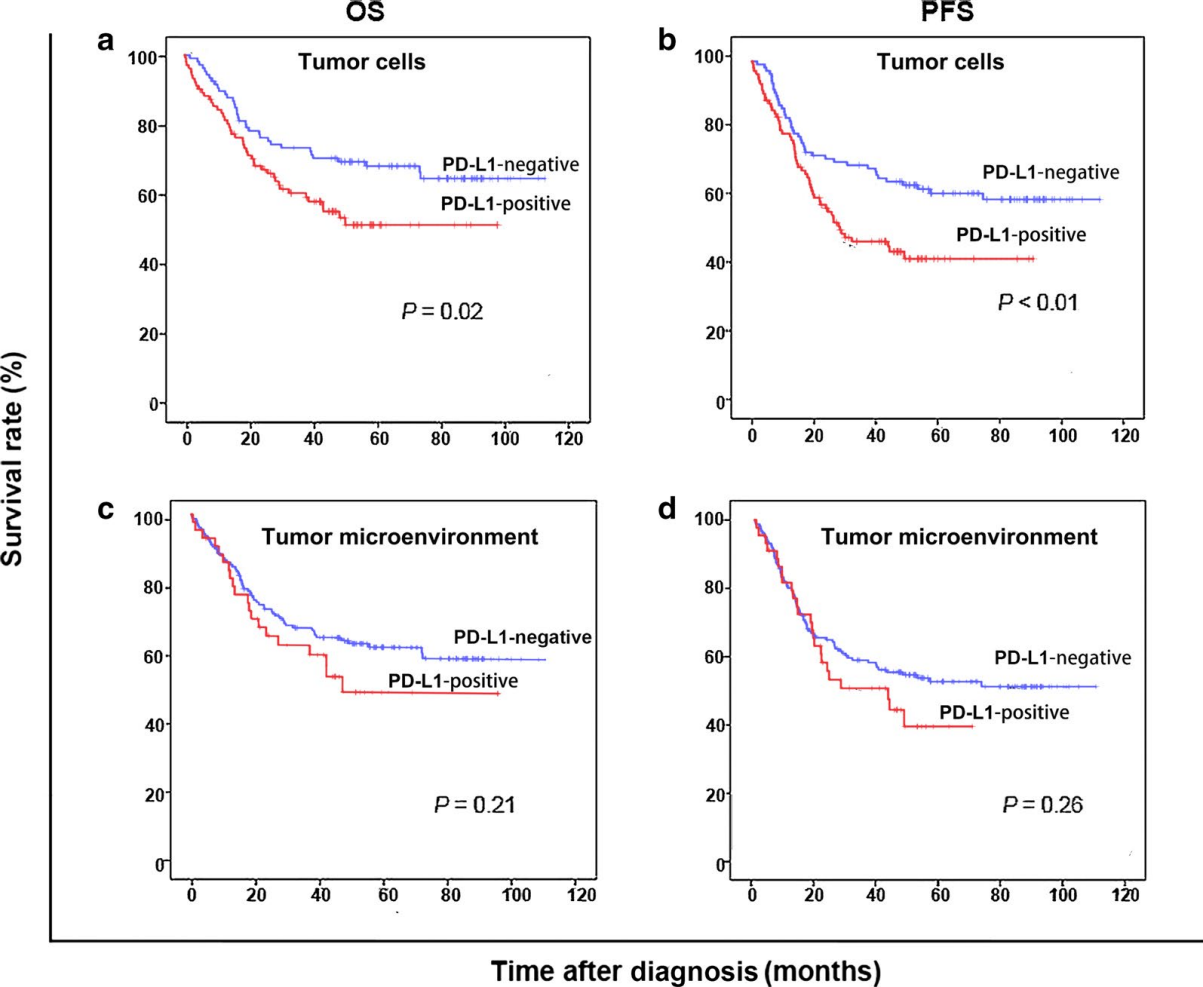
Kaplan-Meier overall survival (OS) and progression-free survival (PFS) curves of patients with diffuse large B-cell lymphoma (DLBCL) according to the expression of programmed cell death-ligand 1 (PD-L1) in tumor cells and microenvironment. **a**, **b** Patients with PD-L1 expression in tumor cells had significantly lower 5-year OS rate (*P* = 0.02) and 5-year PFS rate (*P* < 0.01) compared with patients without PD-L1 expression in tumor cells. **c**, **d** The 5-year OS rate (*P* = 0.21) and 5-year PFS rate (*P* = 0.26) were similar between patients with and without PD-L1 expression in tumor microenvironment

The 5-year OS rate was 59.5%, and the 5-year PFS rate was 50.0% for the 204 DLBCL patients. Patients with PD-L1 expression in tumor cells had significantly lower 5-year OS rate (50.0% vs. 67.3%, *P* = 0.02) and 5-year PFS rate (39.6% vs. 59.6%, *P* = 0.01) than did patients without PD-L1 expression in tumor cells; the 5-year OS and PFS rates were similar between patients with and without PD-L1 expression in tumor microenvironment (Fig. 1).

Univariate analysis showed that age of older than 60, stage III–IV disease, with B symptoms, with spleen involvement, IPI no less than 3, serum lactate dehydrogenase (LDH) level higher than 245 U/L, failure to achieve complete remission (CR) after first-line chemotherapy, and PD-L1 expression in tumor cells were significantly associated with short OS and PFS; β2-MG level higher than 2.52 mg/L and lack of rituximab treatment were only significantly associated with short OS (Table 2). Factors that were significant in univariate analysis were included in the multivariate analysis.

**Table 2:** Univariate analysis of prognostic factors for overall survival (OS) and progression-free survival (PFS) of patients with DLBCL

Variable	5-year OS rate (%)	*P* value	5-year PFS rate (%)	*P* value
Age (years)		< 0.01		0.02
≤ 60	66.7		54.7	
> 60	42.1		38.8	
Clinical stage		< 0.01		< 0.01
I–II	68.2		61.7	
III–IV	50.7		38.4	
B symptoms		0.02		< 0.01
No	63.8		54.2	
Yes	48.7		38.7	
Spleen involvement		0.01		< 0.01
No	62.4		53.0	
Yes	41.8		31.4	
IPI		< 0.01		< 0.01
< 3	64.4		55.6	
≥ 3	41.9		29.6	
LDH (U/L)		< 0.01		< 0.01
≤ 245	72.7		60.1	
> 245	46.2		39.0	
β2-MG (mg/L)		< 0.01		0.07
≤ 2.52	73.2		54.6	
> 2.52	42.2		35.7	
Rituximab		0.04		0.20
Without	53.1		46.4	
With	71.9		58.4	
First-line chemotherapy CR		< 0.01		0.04
No	49.1		45.9	
Yes	72.2		57.2	
PD-L1 expression in tumor cells		0.02		0.01
Negative	67.3		59.6	
Positive	50.0		39.6	
PD-L1 expression in tumor microenvironment		0.21		0.26
Negative	62.0		52.5	
Positive	48.9		39.3	

*IPI* international prognostic index, *LDH* lactate dehydrogenase, *β2-MG* β2-microglobulin, *CR* complete remission, *PD-L1* programmed cell death-ligand 1

In multivariate analyses, age [hazard ratio (HR) 1.38; 95% confidence interval (CI) 1.69–9.40; *P* < 0.01], CR after first-line chemotherapy (HR − 1.54; 95% CI 0.09–0.49; *P* < 0.01), and PD-L1 expression in tumor cells (HR 1.40; 95% CI 1.61–10.23; *P* < 0.01) were independent risk factors for OS; stage (HR 0.69; 95% CI 1.23–3.23; *P* = 0.01) was an independent predictor for PFS (Table 3).

**Table 3:** Multivariate analysis of prognostic factors for OS and PFS of patients with DLBCL

Variate	OS			PFS		
	HR	95% CI	*P*	HR	95% CI	*P*
Age	1.38	1.69–9.40	< 0.01	0.39	0.87–2.51	0.15
Clinical stage	− 0.39	0.22–2.06	0.57	0.69	1.23–3.23	0.01
B symptoms	− 0.93	0.12–1.35	0.63	0.45	0.97–2.56	0.07
Spleen involvement	− 0.05	0.24–3.87	0.95	0.34	0.74–2.67	0.30
IPI	1.01	0.59–12.70	0.20	0.02	0.51–2.04	0.96
LDH	0.29	0.49–3.67	0.57	0.24	0.76–2.15	0.35
β2-MG	0.02	0.33–3.18	0.98	–		
Rituximab	− 0.13	0.34–2.26	0.80	–		
CR after first-line chemotherapy	− 1.54	0.09–0.49	< 0.01	− 0.24	0.48–1.27	0.33
PD-L1 expression in tumor cells	1.40	1.61–10.23	< 0.01	0.46	1.00–2.51	0.05

*IPI* international prognostic index, *LDH* lactate dehydrogenase, *β2-MG* β2-microglobulin, *CR* complete remission, *PD-L1* programmed cell death ligand 1, *–* not included

## Discussion

In the present study, we investigated the expression of PD-L1 in tumor cells and in tumor microenvironment in DLBCL patients. PD-L1 expression in tumor cells was significantly associated with poor prognosis. We also found that PD-L1 expression in tumor microenvironment was associated with resistance to first-line chemotherapy and the expression level of PD-L1 in tumor microenvironment was negatively correlated with that of C-Myc. Furthermore, the non-GCB subtype was associated with PD-L1 expression in either tumor cells or tumor microenvironment.

PD-L1 expression in B-cell lymphomas is uncommon [15]. Generally, low-grade B-cell lymphoproliferative disorders are thought to rarely express PD-L1 [14]. Nevertheless, it was also reported that follicular lymphoma and DLBCL involved several immune escape pathways, suggesting that escape from antitumor immunity was essential in these aggressive lymphomas [42]. Efforts have been made to identify biomarkers of a response to immune checkpoint blockade, in order to identify the subsets of patients who are most likely to benefit from immune checkpoint blockade treatment. Currently, no biomarkers are generally recognized. In the present study, PD-L1 was found to be an independent predictor for OS and the non-GCB subtype was associated with PD-L1 expression in either tumor cells or tumor microenvironment. Besides, PD-L1 expression in tumor cells were associated with high β2-MG and advanced stage, demonstrating its role as an adverse prognostic factor in DLBCL. In another study, PD-L1 expression level was positively correlated with the number of PD-1-positive T cells in activated B-cell-like (ABC)-subtype DLBCL specimens, but was negatively correlated with the number of forkhead box P3 (FOXP3)-positive regulatory T cells in GCB-subtype DLBCL specimens [5]. On the basis of these findings, it may confer that immune evasion owing to PD-L1 expression in tumor cells might be associated with the poor clinical outcomes of patients with ABC-subtype DLBCL. Conversely, the lack of PD-L1 expression in GCB-subtype DLBCL specimens is a plausible explanation for the favorable prognosis associated with this disease subtype [43]. Therefore, PD-L1 expression in tumor cells may be a potential candidate biomarker of response to inhibitors of the PD-1/PD-L1 axis. Immunotherapy targeting the PD-1/PD-L1 pathway may benefit patients with DLBCL, particularly those with non-GCB-subtype DLBCL, which might benefit from blockade of the PD-1/PD-L1 immune checkpoint.

We set the threshold of at least 5% of lymphoma cells with PD-L1 expression for PD-L1 positivity in tumor cells and at least 20% of malignant and non-malignant cells with PD-L1 expression for PD-L1 positivity in tumor microenvironment, conforming to the cut-point used in a previous publication [14]. We observed that 49.0% of patients were PD-L1-positive in tumor cells and 21.6% were PD-L1-positive in tumor microenvironment, which were comparable with those observed in a previous study [42]. Several researchers analyzed the expression of PD-L1 in tumor cells and tumor microenvironment in DLBCL using other standards. Kiyasu et al. [5] defined PD-L1 positivity in tumor cells as over 30% of lymphoma cells staining of both PD-L1 and paired box gene 5 (PAX5); when PD-L1-positive non-malignant stromal cells constituted over 20% of the total tissue from patients without PD-L1 expression in tumor cells, the sample was considered PD-L1-positive in tumor microenvironment. In their study, PD-L1 positivity in tumor cells and tumor microenvironment were observed in 10.5% and 15.3% of DLBCL patients. The higher rates of PD-L1 expression in tumor cells and tumor microenvironment in our cohorts may due to the race and different experimental standards. Fang et al. [43] detected the expression of PD-L1 in tumor tissues from 76 Chinese DLBCL patients and found that PD-L1 was expressed in tumor cells in 26.3% of patients. The smaller sample size in their study may explain the discrepancy.

In the present study on 204 DLBCL patients, PD-L1 expression in tumor cells was an independent risk factor for OS (HR 1.40; 95% CI 1.61–10.23; *P* < 0.01), but not for PFS (HR 0.46; 95% CI 1.00–2.51; *P* = 0.05), in multivariate analysis. These results were in accordance with the findings by Kiyasu et al. [5]. Interestingly, using higher expression levels as cutoff threshold, such as over 30% of lymphoma cells expressing both PD-L1 and PAX5 used by Kiyasu et al. [5] and Xing et al. [44], is more accurate in prognostic prediction than other cutoff values in their studies. In the present study, the differences in survivals could also be distinguished using a cutoff threshold of 5%. Xing et al. [44] retrospectively analyzed data of 84 EBV-negative DLBCL patients and found that patients with PD-L1 expression in tumor cells had a higher proportion of non-GCB-subtype disease than those without PD-L1 expression (71% vs. 30%, *P* = 0.0060). In the present study, PD-L1 expression in tumor cells and in microenvironment were associated with the non-GCB subtype (*P* = 0.02 and 0.04), which typically indicated a poor overall prognosis. The mechanisms responsible for the relationship between PD-L1 expression and poor prognosis are still not clear. In classical Hodgkin’s lymphoma, 9p24.1 amplification [45] and EBV infection [41] are thought to be related to the overexpression of PD-L1. Another study suggested that genetic alterations affecting the PD-L1/PD-L2 locus might lead to the overexpression of PD-L1 [46]. Several studies reported that ABC-subtype DLBCL prominently expressed both PD-L1 and PD-L2 [36, 47, 48]. Genes/pathways expressed in non-GCB-subtype DLBCL showed similarity to those in ABC-subtype DLBCL [49], and the activation of Janus kinase (JAK)/signal transduction and activation of transcription factor 3 (STAT3) signaling might favor the constitutive expression of PD-L1 [50]. This phenomenon suggests that immunotherapies blocking PD-1 and PD-L1 or targeting the JAK/STAT3 signaling pathway may benefit patients with this aggressive subtype of disease.

PD-L1 positivity in tumor microenvironment was found to be associated with resistance to first-line chemotherapy in the present study. The composition and function of tumor microenvironment is an important factor for both immune escape of tumors and antitumoral defense [51]. PD-L1 is known to interact with CD80. CD80 expressed on activated T cells [and possibly antigen-presenting cells (APCs)] can function as a receptor rather than a ligand, delivering inhibitory signals when engaged by PD-L1 [52]. Understanding such complex receptor-ligand interactions in tumor microenvironment will be required to reveal potential immune checkpoint resistance mechanisms [53]. It is possible that small T-cell clones are more important than large T-cell clones in tumor response once effective chemotherapy regimens have significantly debulked the initial tumor load in DLBCL patients [54]. In DLBCL, IHC analysis of tissue biopsies has revealed an association between T-cell infiltration and response to chemotherapy [53]. High CD4^+^ T-cell infiltration has been associated with long OS in DLBCL patients treated with both anthracycline-based regimens and R-CHOP chemoimmunotherapy [55, 56]. Curiel et al. [57] reported that tumor-infiltrating PD-L1-positive myeloid dendritic cells (MDCs) suppressed the induction of interferon-gamma (IFN-γ) in T cells and reduced IFN-γ-positive T cells, indicating that PD-L1-positive MDCs induce T-cell immune suppression in tumor microenvironment. Therefore, the number of PD-L1-positive tumor-infiltrating lymphocytes (TILs) was lower in DLBCL patients with PD-L1 expression tumor microenvironment, which may be due to the suppression of T-cell induction by PD-L1-positive MDCs in tumor microenvironment. High number of PD-L1-positive cells in tumor microenvironment could shield the tumor against attacking TILs [51]. These may be the possible mechanisms underlying why and how PD-L1 positivity in tumor microenvironment impact the treatment resistance.

In the present study, we found that PD-L1 expression in tumor microenvironment had a negative correlation with that of C-Myc (*r* = − 0.20, *P* = 0.04). *C*-*Myc* is a known oncogene in DLBCL. *C*-*Myc* gene translocation is a hallmark of Burkitt lymphoma and was detected in 5%–17% of DLBCL patients [58]. *C*-*Myc* aberrations include gene translocation, gene amplification, and mRNA or protein overexpression. Duranpanteix et al. [59] investigated the role of C-Myc in the regulation of PD-L1 expression in the P493-6 B cell line. They found that the inhibition of C-Myc expression by tetracycline led to an increase in *PD*-*L1* mRNA expression. The regulatory region of *PD*-*L1* gene does not contain a binding site for *C*-*Myc*, whereas *STAT1* and interferon regulatory factor 1 (*IRF1*) directly bind to the promoter of *PD*-*L1* gene to increase its transcription, suggesting that *C*-*Myc* would repress *PD*-*L1* expression at the mRNA level via *STAT1* inhibition [59]. The mechanism of the relationship between C-Myc and PD-L1 expression in tumor environment in the present study needs to be further explored. Casey et al. [60] have shown that *C*-*Myc* regulated the antitumor immune response through binding the promoter region and activating the transcription of *CD47* and *PD*-*L1*, and *C*-*Myc* appeared to initiate and maintain tumorigenesis in a mouse model of *Myc*-induced T-cell acute lymphoblastic leukemia. Inactivation of *Myc* could result in the down-regulation of *PD*-*L1*, thus restoring recruitment of T cells and macrophages and causing tumor shrinkage. Besides, Rossille et al. [61] showed that patients with high levels of serum PD-L1 had a significantly shorter OS than those with low levels of serum PD-L1 within the BCL2-positive population. Therefore, in *Myc*-driven malignancies or even high-grade B-cell lymphomas, especially in those with *Myc* rearrangement, such as double-hit or triple-hit lymphomas, *Myc*- and *PD*-*L1*-targeted combination therapy may be of potential therapeutic benefit.

Several limitations should be noted when interpreting the results of the present work. As a retrospective study, the long time span for the storage of paraffin-embedded samples may affect the results of PD-L1 detection. The missing clinical data may also affect the accuracy of statistical analysis. Therefore, a study with a larger population is needed to verify our findings. It remains unclear whether the expression of PD-L1 is a key factor associated with the clinical prognosis of DLBCL patients treated with PD-1/PD-L1 blockade therapy. Further integration of genomic and clinical data is expected to deepen our understanding of PD-L1 in DLBCL.

## Conclusions

In conclusion, the expression of PD-L1 in both tumor cells and microenvironment is associated with the non-GCB-subtype DLBCL. PD-L1 expression in tumor microenvironment has a negative correlation with the expression of C-Myc, which indicates a role of C-Myc in the regulation of PD-L1 expression. PD-L1 predicts short survival in DLBCL patients. For patients with PD-L1 expression in tumor cells, more strategy such as anti-PD-L1 antibody treatment should be recommended.

## Abbreviations

PD-L1: programmed cell death-ligand 1
DLBCL: diffuse large B-cell lymphoma
ALK: anaplastic lymphoma kinase
EBERs: EBV-encoded RNAs
FISH: fluorescence in situ hybridization
OS: overall survival
